# PROgnosticating COeliac patieNts SUrvivaL: The PROCONSUL Score

**DOI:** 10.1371/journal.pone.0084163

**Published:** 2014-01-02

**Authors:** Federico Biagi, Annalisa Schiepatti, Georgia Malamut, Alessandra Marchese, Christophe Cellier, Sjoerd F. Bakker, Chris J. J. Mulder, Umberto Volta, Fabiana Zingone, Carolina Ciacci, Anna D’Odorico, Alida Andrealli, Marco Astegiano, Catherine Klersy, Gino R. Corazza

**Affiliations:** 1 Coeliac Centre/First Department of Internal Medicine, Fondazione IRCCS Policlinico San Matteo, University of Pavia, Pavia, Italy; 2 Gastroenterology Department, Hôpital Européen Georges Pompidou, Université Paris Descartes, Paris, France; 3 Department of Gastroenterology and Hepatology, VU University Medical Center, Amsterdam, The Netherlands; 4 Coeliac Centre/Department of Clinical Medicine, St Orsola-Malpighi University Hospital, Bologna, Italy; 5 Gastrointestinal Unit, Department of Medicine and Surgery, University of Salerno, Salerno, Italy; 6 Department of Surgical and Gastroenterological Sciences, University of Padua, Padua, Italy; 7 Department of Gastro-Hepatology, AOU San Giovanni Battista Molinette, University of Turin, Turin, Italy; 8 Biometry and Clinical Epidemiology, Fondazione IRCCS Policlinico San Matteo, Pavia, Italy; Centro di Riferimento Oncologico, IRCCS National Cancer Institute, Italy

## Abstract

**Introduction:**

It has been shown that mortality rates of coeliac patients correlate with age at diagnosis of coeliac disease, diagnostic delay for coeliac disease, pattern of clinical presentation and HLA typing. Our aim was to create a tool that identifies coeliac patients at higher risk of developing complications.

**Methods:**

To identify predictors of complications in patients with coeliac disease, we organised an observational multicenter case-control study based on a retrospective collection of clinical data. Clinical data from 116 cases (patients with complicated coeliac disease) and 181 controls (coeliac patients without any complications) were collected from seven European centres. For each case, one or two controls, matched to cases according to the year of assessment, gender and age, were selected. Diagnostic delay, pattern of clinical presentation, HLA typing and age at diagnosis were used as predictors.

**Results:**

Differences between cases and controls were detected for diagnostic delay and classical presentation. Conditional logistic models based on these statistically different predictors allowed the development of a score system. Tertiles analysis showed a relationship between score and risk of developing complications.

**Discussion:**

A score that shows the risk of a newly diagnosed coeliac patient developing complications was devised for the first time. This will make it possible to set up the follow-up of coeliac patients with great benefits not only for their health but also for management of economic resources.

**Conclusions:**

We think that our results are very encouraging and represent the first attempt to build a prognostic score for coeliac patients.

## Introduction

Coeliac disease (CD), a gluten-induced chronic enteropathy, is very common in the Western world [1]. Although its prognosis is excellent in most patients, a few can develop serious complications mainly represented by premalignant and malignant conditions, such as enteropathy associated T cell lymphoma, B cell abdominal lymphoma, refractory CD type 1 and type 2 (RCD1, RCD2), and small bowel carcinoma [2]–[4]. These complications do occur rarely (<1% of CD patients [2], [5]–[8]) but nowadays there is no effective therapy to contrast them and so they dramatically reduce the prognosis of these patients [9]. More precisely, the five-year survival rate is reported to be between 80% and 96% in patients with RCD1, it is between 40% and 58% in patients with RCD2 and it drops to between 8% and 20% in patients with CD complicated by enteropathy associated T cell lymphoma [8], [10]–[14]. So, to develop a tool that allows identification of those coeliac patients at higher risk of complications would be very useful, making it possible to set up the follow-up of coeliac patients according to their specific risk of complications. The patients with a higher risk would be seen much more frequently than patients at lower risk. Consequently, such a tool would not only provide benefits for the health of the patients but it would also help physicians in improving the use of health care resources.

Several studies proved that strict adherence to a gluten-free diet (GFD) is of paramount importance to protect coeliac patients [3]. However, adherence to a GFD can be tested only after it has been followed for a few months. Since complications of CD tend to occur in the first few years after the diagnosis of CD, and the risk then decreases over time [15], an ideal prognostic test should be based on clinical data already available at the time of the initial diagnosis of CD.

Apart from adherence to a GFD, in the last few years other clinical data have been shown to correlate with the risk of developing complications and/or mortality rates of patients with CD. This was shown to be the case for age at diagnosis of CD, time between onset of symptoms and diagnosis of CD (i.e. diagnostic delay for CD), pattern of clinical presentation for CD [15], and HLA-DQ2 homozygosity [16]–[18]. Our aim was therefore to build a prognostic score, based on these very simple clinical characteristics assessed at the time of diagnosis of CD, that will identify those coeliac patients at the greatest risk of developing complications.

## Results

On the basis of the above mentioned enrolment criteria, we obtained clinical data from 116 cases (patients with complicated CD; 74 F, mean age at enrolment 55±14 yrs, 50 dead) and 181 controls (coeliac patients without any complications; 116 F, mean age at enrolment 53±15 yrs, all alive after a mean follow-up of 69 months, median 46, range 1–381). Unfortunately, genomic HLA was available in only 85 cases and 120 controls. Table 1 shows the complications found in the cases and how many patients died of them. As expected, enteropathy associated T cell lymphoma was the most common and most serious one. The Pavia centre provided data for 35 cases and 54 controls; Paris provided data for 31 cases and 34 controls; Amsterdam 20 cases and 38 controls; Bologna 15 cases and 26 controls; Naples 10 cases and 20 controls; Turin 3 cases and 6 controls; Padua 2 cases and 3 controls.

**Table 1:** Type and number of complications found among the 116 cases and number of cases who died because of either the complication or other unrelated causes.

Type of complication	N. of complications	N. of cases dead due to complications	N. of cases dead due to other causes
**RCD1**	39	3 (7%)	4 (57%)
**RCD2**	32	4 (9%)	2 (29%)
**EATL**	42	33 (77%)	0 (0%)
**SBC**	15	3 (7%)	1 (14%)
**Total**	128	43	7

One case with RCD1 and eleven cases with RCD2 developed EATL. RCD1: refractory coeliac disease type 1; RCD2: refractory coeliac disease type 2; EATL: enteropathy associated T cell lymphoma; SBC: small bowel carcinoma.

Table 2 shows the analysis of predictors in cases and controls. Diagnostic delay for CD, prevalence of classical presentation for CD and homozygosity for DQ2 were found to be significantly different between cases and controls. Only age at diagnosis of CD did not differ. We noted, however, that only 7% of cases were younger than 30 when found to be affected by CD and 74% of them were older than 40 when found to be affected by CD.

**Table 2:** Analysis of the differences between predictors found in cases and controls.

	CASES	CONTROLS	STATISTICS
**Age at diagnosis of CD (mean ± SD)**	49 years ±15	48 years ±15	T test, p = 0.26 ns
**Diagnostic delay for CD** *	9 months, IQR 3–26	18.5 months, IQR 4–75	Chi^2^, p = 0.026
**Classical presentation of CD**	83/115 (72%)	75/181 (41%)	Chi^2^, p<0.0001
**HLA-DQ2 homozygosity** **	37/85 (43%)	28/120 (23%)	Chi^2^, p = 0.0023

Diagnostic delay was applicable in 81 cases and 104 controls;

genomic HLA was available for 85 cases and 120 controls. CD: coeliac disease; DS: standard deviation; IQR: interquartile range.

**Figure 1:**
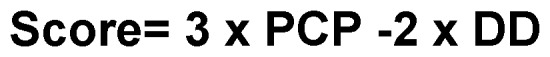
Formula to obtain the PROCONSUL score. PCP = pattern of clinical presentation: assign 0 if non classical/asymptomatic and 1 if classical; DD = diagnostic delay: assign 0 if <6 months and 1 if >6 months.

**Figure 2:**
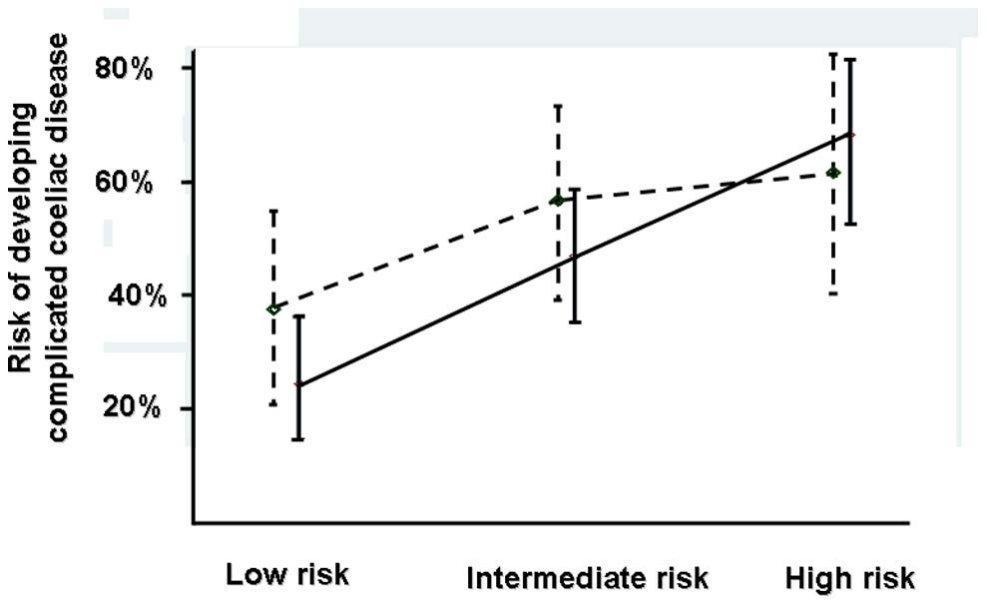
Analysis of tertiles based on the conditional logistic models. (mean, 95% confidence intervals). Black lines show analysis without HLA-DQ2 homozygosity (Chi square, p<0.001) while dotted lines show analysis including HLA-DQ2 homozygosity (Chi square, p = 0.005).

The conditional logistic model performed on the basis of the clinical predictors found to be significantly different between cases and controls (i.e. pattern of clinical presentation and diagnostic delay) obtained a formula that made it possible to calculate the prognostic score (fig. 1). The subsequent tertiles analysis showed a statistically significant relationship between score and risk of developing complications (fig. 2). Table 3 shows the results of our score and the relative risk of developing complications, in a more practical and easy to use way.

**Table 3:** A practical and easy system to calculate the prognostic score in patients with coeliac disease.

Pattern of clinical presentation	Diagnostic delay	Result of the score	Risk of complication
**Non classical/asymptomatic**	>6 months	−2	Low
	<6 months	0	Low
**Classical**	>6 months	1	Intermediate
	<6 months	3	High

The results given by the conditional logistic model performed on the basis of not only the clinical predictors (i.e. pattern of clinical presentation and diagnostic delay) but also HLA DQ2 homozygosity were not as good as the one without HLA (fig. 2).

## Discussion

In medicine, there are several conditions routinely evaluated by means of diagnostic and prognostic scores. In gastroenterology, this is the case for the Crohn’s Disease Activity Index, the Glasgow score for pancreatitis, the Child Pugh score for liver insufficiency, and the Rockall score for gastrointestinal bleeding. All these scoring systems are very useful but they are far from being perfect. Some of them have been modified several times in the attempt to achieve a better performance and, on the other hand, some pathological conditions are the focus of different scores, which clearly show that the best one has not yet been found.

In this study we developed for the first time a three-level numeric score (low, intermediate, high) that calculates the risk for a newly diagnosed coeliac patient of developing complications. Nowadays, there is still no agreement on how to organise the follow-up of coeliac patients in everyday clinical practice [21]. Some authorities suggest performing a duodenal biopsy in all adult patients; others think that a clinical and a serological follow-up is sufficient in most of them and prefer to repeat a duodenal biopsy only when complications are suspected. Although our results need to be confirmed prospectively, our score could be of help in organising the follow-up of newly diagnosed coeliac patients. Patients at higher risk could be closely followed up, not only from a clinical point of view but also a histological one. On the other hand, patients at lower risk could be followed up less frequently and maybe only from a clinical point of view, without the need to perform a second duodenal biopsy. This could have obvious benefits regarding not only the health of the patients but also the management of health care resources.

A strict GFD is well known to be of paramount importance in protecting coeliac patients from malignancies. Our choice of not using GFD adherence as a predictor could therefore seem a debatable one. It is, however, understandable if we keep in mind that 50/116 cases were already dead when the study was performed. Moreover, the median time between diagnosis of CD and diagnosis of complications was very short (20 months). Again, the median time between diagnosis of complications and death was a mere 11 months, and the 25^th^ percentiles were 3.25 and 2 months, respectively. Taking into account that compliance with a GFD can be tested only after no fewer than 6–12 months, we think that an efficient prognostic tool cannot afford to waste such a vital amount of time. Therefore, we feel that only a prospective study can test how GFD adherence can be used as a predictor in a prognostic score for CD. Finally, since the importance of a strict GFD for CD patients is obviously unquestionable, we were worried that including an evaluation of the compliance with GFD in the scoring system could have the deleterious effect of reducing diet adherence in those coeliac patients with a good prognostic score.

To develop our scoring system, we utilised four predictors represented by age at diagnosis of CD, diagnostic delay for CD, pattern of clinical presentation, and HLA typing. These were not the only possible predictors since other parameters are also known to correlate with mortality. However, they are certainly the simplest ones to use in clinical practice and their correlation with risk of complications has been shown by more than one study [15]–[17]. As expected, both the classical pattern of clinical presentation and DQ2 homozygosity were more frequent among cases. Conversely, diagnostic delay gave a totally unexpected result. On the basis of previous studies [15]–[17], we thought that diagnostic delay would have been longer in cases rather than controls but it turned out to be exactly the other way around. To explain this finding, we must not forget that a Swedish study showed no relationship between mortality and diagnostic delay in coeliac patients [22]. We also have to underline that all the studies that showed a relationship between diagnostic delay and mortality of coeliac patients were actually population studies that compared mortality of coeliac patients with mortality of the general population. The present study, on the other hand, is a case-control study where the diagnostic delay of complicated coeliac patients (cases) was compared with the diagnostic delay of uncomplicated coeliac patients (controls). Finally, to explain this finding, we could hypothesise that, in those coeliac patients who developed complications, the complication itself had already been triggered when CD was diagnosed. The course of the disease was much more aggressive and so it was easier and faster to reach a diagnosis.

As far as HLA typing is concerned, we underlined in the results that genomic HLA was available in only 85 cases and 120 controls. A selection bias was also very likely to exist. According to our conditional logistic model, not performing HLA typing had a strong protective effect (data not shown). This means that the more serious the patient was, the more likely he/she was to undergo HLA typing. So, taking into account not only this selection bias but also the costs of HLA typing and, most important, the unsatisfactory results we obtained including HLA in the model, we believe that the score based only on diagnostic delay and pattern of clinical presentation is the most suitable one (table 3).

Probably because of the recruitment criteria, age at diagnosis of CD was identical in cases and controls and could not be used in the model. Since age at diagnosis of CD was shown to correlate with mortality of coeliac patients [2], [15], this can certainly be considered to be a limit of our study. However, we noted that 74% and 53% of the cases had been found to be affected by CD after the age of 40 and 50, respectively. On the other hand, only 7% of the cases had been found to be affected by CD before the age of 30. We think that this confirms that age at diagnosis of CD correlates with the risk of developing complications. It could also suggest that the complications of CD should be taken into account only in adult patients while in younger patients the risk is negligible.

Although a prospective study would probably allow GFD adherence to be used as a predictor, organising this study prospectively would be a very hard task. If we assume that complicated CD occurs in less than 1% of all coeliac patients [2], in order to collect the 100 cases required by our sample size definition, at least 10000 adult patients would need to be enrolled at the time of diagnosis of CD. They would then need to be followed up for at least a few years before it would be possible to distinguish between cases and controls. Moreover, five other prestigious Italian and European centres did not take part in this study because they could not provide us with the required data. This further underlines how difficult it was to perform this study. The second problem is the lack of standardized diagnostic criteria for the complications of CD. Although this problem is unlikely to influence the diagnoses of enteropathy associated T cell lymphoma and small bowel carcinoma, it could affect the diagnoses of RCD2 and, consequently, those of RCD1. Discrepancies in the diagnostic criteria for complications of CD have already been described [6]. Having designed a multicentre study to achieve the required sample size, we could not avoid this issue.

## Methods

### Design of the Study

To identify predictors of complications in patients with CD, we organised an observational multicenter case-control study based on a retrospective collection of clinical data.

### Cases and Controls

In this article, the term “cases” indicates patients with CD who subsequently developed a complication while the term “controls” indicates patients with CD who did not subsequently develop a complication. Cases were recruited among patients found to be affected by CD in adulthood (age >18 years) and that later developed any of the following complications: RCD1, RCD2, enteropathy associated T cell lymphoma, small bowel carcinoma. Diagnoses of enteropathy associated T cell lymphoma and small bowel carcinoma were based on histological criteria; diagnosis of RCD2 was based on a flat duodenal mucosa not responding to 12 months on a GFD and evidence of an aberrant intraepithelial lymphocyte population consisting of intraepithelial lymphocytes lacking surface CD3, CD4 and CD8 and/or gamma chain T cell monoclonal rearrangement; diagnosis of RCD1 was based on a flat duodenal mucosa not responding to 12 months on a GFD but without the diagnostic criteria for RCD2 [10]–[12].

Controls were recruited among patients found to be affected by CD in adulthood (age >18 years) on the basis of a flat duodenal biopsy and positive endomysial/tissue transglutaminase antibodies while on a gluten-containing diet. They did not develop any of the above mentioned complications. Controls were seen in the same year and at the same outpatient clinic as the cases and were matched to them by gender and age at enrolment (±5 years). Age at enrolment for cases was the age at diagnosis of the CD-complicating condition; for controls it corresponded to the age at the last time they were seen in the clinic. Two, or at least one, matched controls were enrolled for each case.

HLA-DQA1 and DQB1 gene polymorphism was analyzed using PCR-SSP and/or revPCR-SSO techniques.

### Data Collection

Date of birth, date of diagnosis of CD, gender, date of enrolment, pattern of clinical presentation of CD (classical: patients complaining of diarrhoea and/or weight loss; non classical: patients not complaining of diarrhoea and weight loss; asymptomatic: patients without any symptoms and diagnosed thanks to serological screening [19]), diagnostic delay of CD (months between onset of symptoms leading to diagnosis of CD and diagnosis itself; if the patient was found to be affected by CD because of familiarity and complained of no symptoms, diagnostic delay was not applicable), genomic HLA typing (homozygosity/heterozygosity for DQ2/8), type of complication, and date and cause of death (if the subject is still alive, date of the last time he/she was seen in the clinic) are the very simple data we collected for each case and each control. Data were collected with an ACCESS stand-alone electronic database specifically developed in Pavia.

### Ethics Statement

Since many cases had already died when their clinical data were collected, it was not possible to obtain signed consent. Therefore, after verifying the good quality of the data, they were all irreversibly anonymized. This was specifically waived by the ethics committee of the Fondazione IRCCS Policlinico San Matteo that approved the study according to the Declaration of Helsinki.

### Statistics

#### Sample size definition

To build a prognostic score, 10 events per predictor need to be included. A total of 10 candidate predictors were identified from the literature [20]. Thus a minimum of 100 cases must be enrolled, together with 200 controls, for a total of 300 patients (200 if only 1 control per case is available).

#### Statistical analysis

The prognostic score was built by means of a conditional logistic model (conditioned on the matching criteria) including all the variables collected via the ACCESS database. The coefficients derived from the prognostic index were rounded to the closest 0.5 for computation of the score. Stata 11 (StataCorp, College Station, TX, USA) was used for computation.

## Conclusions

Our results are very encouraging and represent the first attempt to build a prognostic score for coeliac patients.
